# *Lacticaseibacillusparacasei* BNCC345679 revolutionizes DSS-induced colitis and modulates gut microbiota

**DOI:** 10.3389/fmicb.2024.1343891

**Published:** 2024-03-27

**Authors:** Waqar Ahmad, Ahmad Ud Din, Taj Malook Khan, Mujeeb Ur Rehman, Adil Hassan, Tariq Aziz, Metab Alharbi, Jianbo Wu

**Affiliations:** ^1^Basic Medicine Research Innovation Centre for Cardiometabolic Diseases, Ministry of Education, Southwest Medical University, Luzhou, China; ^2^Plants for Human Health Institute, Department of Food, Bioprocessing and Nutrition Sciences, North Carolina State University, Kannapolis, NC, United States; ^3^Department of Pharmacy, Faculty of Biological Sciences, Quaid-i-Azam University, Islamabad, Pakistan; ^4^Laboratory of Nano/Micro Composite Materials and Devices, Chongqing University of Science and Technology, Chongqing, China; ^5^Department of Agriculture, University of Ioannina, Ioannina, Greece; ^6^Department of Pharmacology and Toxicology, College of Pharmacy, King Saud University, Riyadh, Saudi Arabia; ^7^Key Laboratory of Medical Electrophysiology, Ministry of Education, Institute of Cardiovascular Research of Southwest Medical University, Luzhou, China; ^8^Laboratory for Cardiovascular Pharmacology, Department of Pharmacology, School of Pharmacy, Southwest Medical University, Luzhou, China

**Keywords:** DSS-induced colitis, dysbiosis, microRNA, gut microbiota, gut inflammation, probiotics, *Lacticaseibacillus paracasei* BNCC345679

## Abstract

The gut microbiota plays an important role in the disease progression of inflammatory bowel disease. Although probiotics are effective against IBD, not many studies have investigated their effects on gut microbiota composition and immunomodulation in mouse colitis models. Our study aimed at the therapeutic effects of *Lacticaseibacillus paracasei* BNCC345679 for the first time and explored its impact on gut microbiome dysbiosis, inflammatory cytokines, related miRNAs, VCAM-1, oxidative stress, intestinal integrity, and mucus barrier. We found that oral intervention of *L. paracasei* BNCC345679 affects recovering beneficial microbial taxa, including *lactobacillus* spp. and *akkermansia* spp., followed by improved body weight, DAI score, and inflammatory cytokines. *L. paracasei* BNCC345679 mitigated oxidative stress and increased the expression of intestinal integrity proteins MUC2 and ZO-1. These results suggested that *L. paracasei* BNCC345679 has the capacity to reduce DSS-induced colitis and has the potential as a supplement for the mitigation of IBD.

## 1 Introduction

IBD is a set of inflammatory gut disorders and is a worldwide complication, specifically affecting those who consume an excessive Western diet. However, underlying etiology and complete disease pathogenesis remain elusive. Genetic and environmental factors, eating habits, abnormal intestinal barrier, and immunological functions, as well as gut dysbiosis, are some of the main factors thought to be involved in the initiation and disease recurrence (Molodecky et al., 2012). One of the most frequently employed *in vivo* models for ulcerative colitis is dextran sulfate sodium (DSS)-induced colitis in mice, characterized by increased permeability, leading to the disruption of the mucous layer, increased levels of immunological molecules, and subsequently abnormal epithelial barrier function (Alemany-Cosme et al., 2021; Haneishi et al., 2023).

Gut microbiota has a well-recognized and pivotal part in the recurrence and pathogenesis of UC. Dysbiosis and disturbances in the gut disrupt the host and microbes’ interaction, causing changes in the colonic epithelium and promoting inflammation by regulating cytokine activity, leading to disease susceptibility. UC and reduced gut microbiota diversity have a notable association with each other. Moreover, critical commensal or symbiotic species are necessary to maintain immune homeostasis. Despite the clear association between UC and the gut microbiome, the causative or consequential relationship between dysbiosis and the disease remains unclear (Buttó and Haller, 2016). One probable mechanism is that the primary damage of the crucial intestinal epithelial barrier allows opportunistic invasive bacterial species. Consequently, stimulation of the immunological responses occurs which leads to inflammation and later to enormous intestinal tissue damage or colitis (Guenther et al., 2016). This initiation of inflammation and colon tissue destruction can then result in the activation of utilizing combined non-specific and specific immune systems, and the body releases adhesion molecules, resulting in flaring up the inflammatory response. Dysbiosis may also cause oxidative stress, host defense modulation, and increased or decreased production of bacterial metabolites produced due to dysbiosis. Oxidative stress is one of the key indicators of UC. The lack of antioxidants leads to intestinal damage and inflammation by increasing pro-inflammatory factors (Vargas Robles et al., 2016).

Additionally, microRNAs are associated with the gut microbial populations and are considered crucial players in IBD pathogenesis and thus associated with IBD and colitis (Wang and Chen, 2019). These miRNAs exhibit functions as essential factors and regulators of colonic infection, influencing the immune response and mucus barrier. It is assumed and has been previously investigated that some probiotics regulate the effect of microbial signals on the immune response by regulating the expression of associated miRNA. Most likely, strategies based on targeting the microbiota may lead to implied immunomodulatory effects (Algieri et al., 2021).

Recently, many studies have presented the effects of different probiotics in both animal models and humans because they are considered safe and can be well tolerated, if used adequately. The results have revealed their effects on maintaining normal intestinal flora and immunomodulatory activity (Zhang et al., 2019). *Lactobacillus* spp. are one of the frequently used probiotics for gastrointestinal disorders. They have verified efficacy, genetic stability, host safety, and the ability to sustain their survival while passing through the gastrointestinal tract (Jang et al., 2019). For instance, *L. casei*, *L. paracasei*, and *L. rhamnosus*, among other several strains, are widely recognized and have been used as safe probiotics in many food and agriculture-related products because of their promising health promoting properties (Hill et al., 2018). Moreover, the efficacy of probiotics to fine-tone human health is strain-specific (Gill and Guarner, 2004) where our focused strain has yet to be previously investigated for its efficacy to colitis. There is still no specific knowledge about the effect of *L. paracasei* BNCC345679 in the amelioration of colitis through the modulation of gut microbiome and associated miRNAs. Considering these limitations, our study objective was to examine the intestinal anti-inflammatory properties and possible mechanisms of *L. paracasei* BNCC345679 in a murine DSS model of colitis. In this study, we have focused on regulating the probiotic role of *L. paracasei* BNCC345679, with genes associated with redox imbalance, redness, and gut zonula occludens proteins. We further evaluated the impact of the strain on the gut microbiome and the immune response, with a special spotlight on the five selected miRNA expressions associated with colitis.

## 2 Materials and methods

### 2.1 Sample preparation

*Lacticaseibacillus paracasei* BNCC345679 was obtained from the General Microbiological lab China and isolated from fermented probiotic yogurt. It is a facultative aerobe having the growth time of 72 h and optimum temperature of 37°C. Colony structure was humid, round, convex-shaped, opaque colored, with the size of 1–2 mm. To activate the bacterial strains, the well-known medium MRS accompanied with 0.5 g/L L-cysteine (Shanghai Yuanye Biotechnology Co., Ltd.) was used to culture the lyophilized stock, which was suspended initially and diluted in PBS. The culture was grown in an anaerobic environment and incubated. The bacterial broth was subjected to centrifugation. To prepare the oral dosage, the residual media from the centrifuged bacterial broth were removed and swept three times with PBS, and then, the pellets were re-suspended for repeated dosages every day. The colony-forming unit (CFU) was measured and optimized for oral delivery using the colony counting technique. A volume of 0.2 mL of suspension was orally gavage daily to the mice; the suspension contains approximately 10^9^ CFU/mL of the bacterial strain in PBS.

### 2.2 Probiotic treatment of animals

In this study, 21 male C57JBL/6 mice (49 days old) were used. The mice were supplied an *ad libitum* normal diet and autoclaved water for 1 week to enable them to adapt to the new environment. The mice were housed at 25°C animal rooms with half a day in brightness and the other half in darkness. Later on, mice were characterized into three clusters, i.e., control, DP, and DSS (DSS + *L. paracasei*), (*n* = 7), housed in individual cages per group, and given the applicable treatments. The normal group received autoclaved water and oral gavage of 0.2 mL daily PBS, while the DSS group received 3% DSS salt dissolved in drinking water together with 0.2 mL of PBS. The DP group had access to 3% DSS dissolved in drinking water and were daily gavage with 0.2 mL of approximately 10^9^ CFU/mL *L. paracasei* BNCC345679. Oral gavage was administered for the duration of approximately 4 weeks, whereas DSS regime was from 3 weeks to 4 weeks straight for 7 days. The mice were weighed every morning throughout the DSS treatment, and their disease severity was recorded through an adopted disease activity index (DAI) score.

### 2.3 Colitis induction and evaluation

Mice were subjected to 3% (w/v) DSS salt for 7 days to induce acute colitis (Wirtz and Neurath, 2007). In brief, 3% DSS were added to the drinking water, and mice were allowed to have it. A freshly prepared DSS solutions were given to the mice on a daily basis. Mice were observed daily, and DAI was evaluated frequently during DSS administration using a modified disease scoring system which includes stool consistency, weight loss, and hematochezia. An occult blood kit was used to detect blood in the feces. Supplementary Table S1 shows standards for the scoring scale. The mice were euthanized, and blood was collected and stored for cytokines and other related parameters analysis. Colon was carefully isolated from each mouse, measured, and photographed. The fecal contents were carefully collected and kept at −80 Degree Centigrade for follow-up microbial diversity analysis. The animal study was reviewed and approved by the Bioethical Committee of Southwest Medical University, Luzhou, under the approval number: SWMU 202/0049.

### 2.4 Staining

The gut system is a protected covering of a mucous layer known as glycoprotein, which is released by secretory granules of mucous cells present in the intestinal crypt. These granules and mucin glycoprotein, of which MUC2 is the primary indicator produced, were detected through Alcian blue by staining mucin contents. Alcian blue staining was used to see the amount of disruption in the colonic mucosal surface, as reported by Steedman (1950).

### 2.5 Evaluation of MPO, MDA, and SOD

The efficiency of a crucial indicator for inflammatory cell infiltration, myeloperoxidase (MPO), oxidative stress parameter malondialdehyde (MDA), and antioxidant enzyme superoxide dismutase (SOD) in the colon was assessed with a locally available kit, and the manufacturer’s advices were followed for quantification (Nanjing Jiancheng Co., Ltd., Nanjing, China). In brief, the colon from the mouse was stacked and cut into smaller parts in ice-cold buffer in a ratio of 1:10 w/v. The colonic tissue homogenization was made in consonance with the manufacturer guidelines using “IKA, Staufen, Germany.”

### 2.6 Histological assessment of colon tissues

The colon tissue specimens were first embedded in paraformaldehyde; later sections were prepared and then dyed with eosin and hematoxylin. NIKON Eclipse Ci was used to take images for histological investigation. An adopted scoring system was used to establish the seriousness of each mouse’s colonic histological injury (n = 7) based on the extent of inflammation, percent area, crypt damage, intensity of mucosal layer damage, and variety of abnormal examination (Supplementary Table S2).

### 2.7 Quantitative real-time polymerase chain reaction

From RNA extraction to qRT-PCR, the procedure was performed as done by Din et al. (2020). In brief, RNA was isolated from the samples and then quantified, followed by synthesis of cDNA, according to the kits manufacturer’s provided directions. The primers employed in this study are given in Supplementary Table S3.

Then, qPCR miRNA kit was used to quantify the five types of miRNA as per manufacturer protocol, which are also described by Din et al. (2020).

### 2.8 Immunofluorescence staining

IF was done on a paraffin-fixed colon slice (7um). The slides were rinsed with PBS and left on a benchtop for 10 min before being submerged in 1% sodium citrate solution and later removed and subjected to xylene wash for paraffin removal and antigen retrieval. For the purpose of fluorescence, the slides were blocked with 1% bovine serum albumin (BSA, Sigma) for 60 min followed by incubation with the primary antibody at a dilution of 1:501 and washed with PBS three times, 5 min each, and afterward, the samples were incubated with goat anti-rabbit Alexa 594, goat anti-mouse Alexa 488, or secondary donkey anti-rat Alexa 488. After subsequent PBST washes for the removal of unbound antibodies, the samples were then dyed with DAPI for 15.0 min to achieve nuclear staining. At the end, the added mounting medium and the tissue area were veiled with a cover slip, and the sides were closed with nail polish to keep it hydrated. A Leica confocal microscope was used to visualize the tissue samples and acquire images. Image J was utilized to analyze and quantify the images and protein intensity from each mouse tissue (*n* = 7).

### 2.9 Cytokine analysis

A 50 mmol/L solution of ice-cold PBS solution (1:9, w/v) was used to homogenize the colonic samples. After centrifugation of the homogenate, the supernatant solution was gathered for further procedure. The content of protein was determined, and the results were adjusted to the tissue protein expression level. The cytokines were measured by enzyme-linked immunosorbent assay (ELISA). IL-1β and TNF-α concentrations in the mouse’s colon were measured utilizing a commercially accessible kit (Fankewei TNF-α, LOT Number: 202108 and IL-1β Beyotime, Catalog Number: PI301) conducted as per company’s protocol.

### 2.10 Extraction and blot analysis

The amino acid content in gut tissue cocktails was evaluated. Protein extraction was performed using native lysis buffer, and its amount was determined using a BCA kit. In the same way, 50 μg of protein were loaded onto an sodium dodecyl-sulfate polyacrylamide gel electrophoresis (SDS-PAGE) gel and then relocated to membranes. Immersed in fat-free milk in 120 days, the membranes underwent blotting with two particular primary antibodies, Histone H3 (1:500) and P65, which were diluted in 3.0% BSA and 0.10% Tween 20/buffer solution and were then incubated. The blot was incubated with secondary anti-mouse IgG after being washed three times with TBS (1,2000). The chemiluminescence kit was used to observe the bands (Bio-Rad). Once the protein expression was measured using Alpha Ease FC, the obtained blots were meticulously organized and labeled.

### 2.11 Computational study to decode gut microbiome

The fecal samples were collected and kept in an −80°C refrigerator until the DNA could be extracted using Omega Bio-Tek, Inc., (GA 3007, USA).

The total genomic DNA was extracted, and by loading on 1.2% agarose gel electrophoresis, assessment of DNA concentration and purity was carried out to ensure accurate quantification and quality of the extracted DNA samples. The DNA that was extracted underwent dilution using sterile water to attain a concentration of 1 ng/ μl. For the amplification of the 16S rRNA, a particular primer with a barcode was used. For the PCR analysis, the Phusion High-Fidelity PCR Master Mix from New England Bio-Labs was combined with 0.2 M forward and reverse primers and roughly 10 ng of template DNA in a 30 L reaction system. The thermal cycling protocol was devised to execute a total of 30 cycles, beginning with an initial denaturation phase at a temperature of 98°C for a duration of 10 s. This was followed by an annealing step at 50°C for 30 s and then an elongation step at 72°C for 60 s. The final step was an extension at 72°C for a period of 5 min. For detection and visualization, PCR products were combined with 1 × volume of loading buffer that included SYB green. The mixture was then electrophoresed on a 2% agarose gel. For further experiments, the samples exhibiting a band between 400 and 450 bp were selected for further analysis. According to Li et al. (2017) instructions, library construction, sequencing, and bioinformatics analysis were completed. Bioinformatics analysis was carried out as per the guidelines and procedures on the Majorbio Cloud platform.^1^

### 2.12 Biostatistics

Statistical analysis was carried out using GraphPad Prism ver.8.0.1 (GraphPad Software Inc., CA, United States). Analysis was determined through one-way analysis of variance (ANOVA), and Tukey’s post-hoc test was performed to compare the groups with the non-colitic group. Data with a *p*-value <0.05 were taken statistically significant. The degree of significance was indicated by the notation *p = 0.05, **p = 0.001, and ***p = 0.0001, while the absence of any notation indicated lack of statistical significance.

## 3 Results

### 3.1 *Lacticaseibacillus paracasei* BNCC345679 alleviates the symptoms of DSS-induced colitis

**Figure 1:**
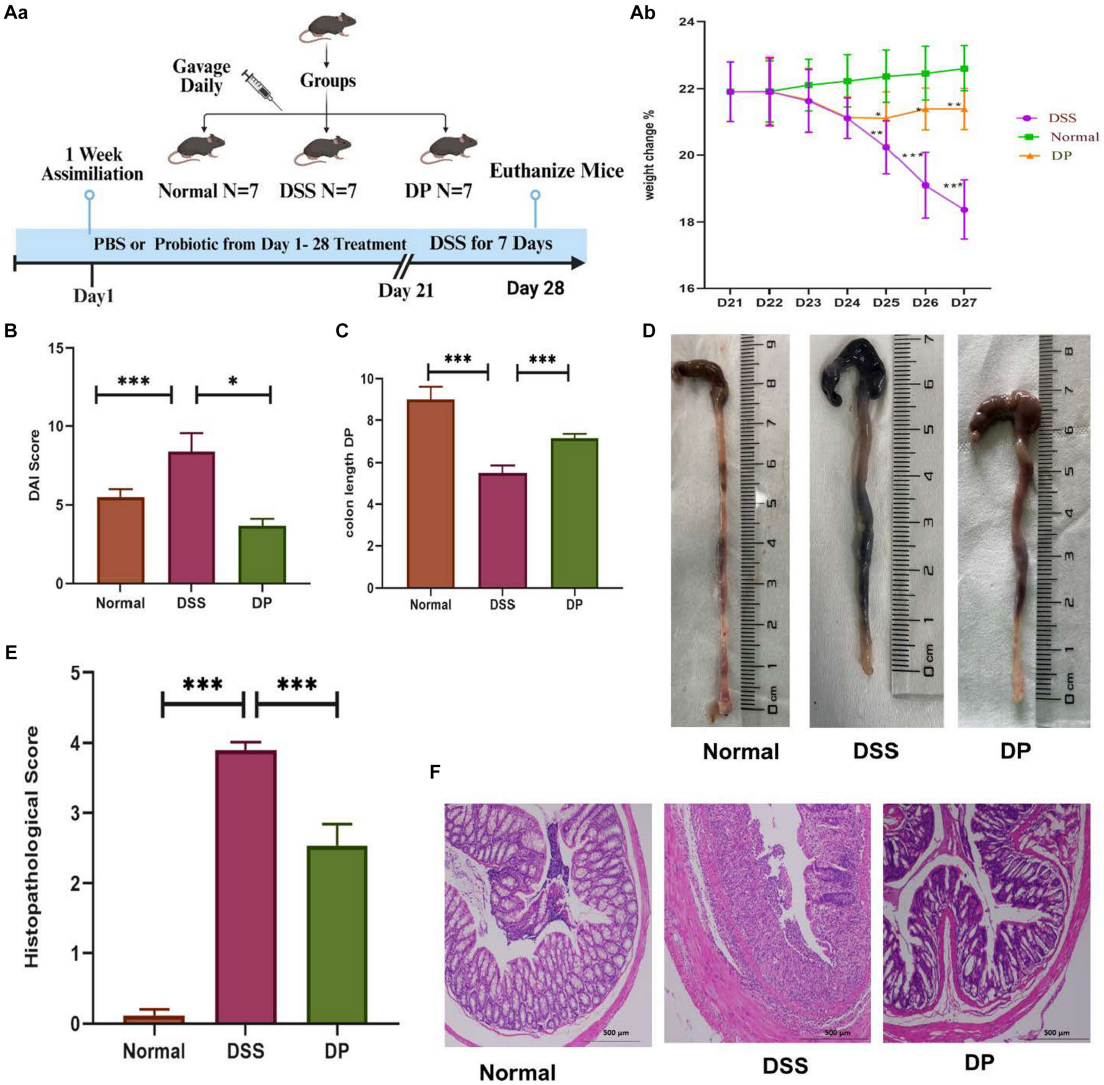
Attenuation of clinical manifestations in DSS-induced colitis by *L. paracasei* BNCC34567 **(Aa)** division of groups showing 1-week assimilation time followed by 21 days of water, 3% DSS, and 3% DSS + probiotic treatment leading to euthanization of mice on 28th day. **(Ab)** Body weight percentage change relative to original weight prior to the induction of DSS induction. Body weight was recorded daily throughout the experiment. **(B)** DAI assessment in DSS-induced colitis, and the entire colon was collected on day 7. **(C,D)** Colon length was measured. This was quantified as disease severity indicator. **(E)** Measurement of the histological score is as per (Supplementary Table S2). **(F)** Histopathological assessment of colonic inflammation. H/E staining was carried out to check disease severity in the distal portion of colon. Scale bar: 500 μm. The data were subjected to analysis of variance (ANOVA) to determine the statistical significance. The degree of significance was indicated by the notation *p = 0.05, **p = 0.001, and ***p = 0.0001, while the absence of any notation indicated lack of statistical significance (*n* = 7). DSS, dextran sulfate sodium; DAI, disease activity index; H/E, hematoxylin and eosin.

The impact of *L. paracasei* BNCC345679 was examined in a mouse DSS-induced colitis model. The DSS model group exhibited a significant lower weight, commencing from the third day and by day 7, as contrast to the normal group. The mice in the DSS + *L. paracasei* BNCC34567 (DP) treatment group showed a significant decline in weight compared to normal after the fourth day but continued to maintain their weight until the seventh day of experiment. However, the weight reduction was much less severe than the model group (Figures 1Aa,b; *p* < 0.005).

Compared to the normal group, the disease activity index (DAI) score of the DSS model group raised significantly, indicating the onset of colon inflammation, while DAI score of the DP group lowered attributing the amelioration of disease severity significantly (Figure 1B).

Colon length varied significantly between the three groups; for instance, colon of the normal group was the longest one, i.e., 8.5 cm. However, the colon length was shortened to 5.5 cm in the DSS model group, while the length of the colon after DP treatment was 6.8 cm (Figures 1C,D).

Histopathological analysis revealed no signs and symptoms of any disease and inflammation in the colon stained with H/E in the normal group. On the other hand, the DSS model group showed highest pathological score showing significant inflammation and cellular infiltration, crypt and goblet cell deterioration, and epithelial cell disruption. The DP-treated group showed mild signs of colitis with notable reduction in goblet cell loss. These results exhibited the watchful role of *L. paracasei* BNCC34567 in mitigating DSS-induced colitis (Figures 1E,F).

### 3.2 *Lacticaseibacillus paracasei* BNCC345679 exhibits antioxidant and anti-inflammatory properties in the colon

**Figure 2:**
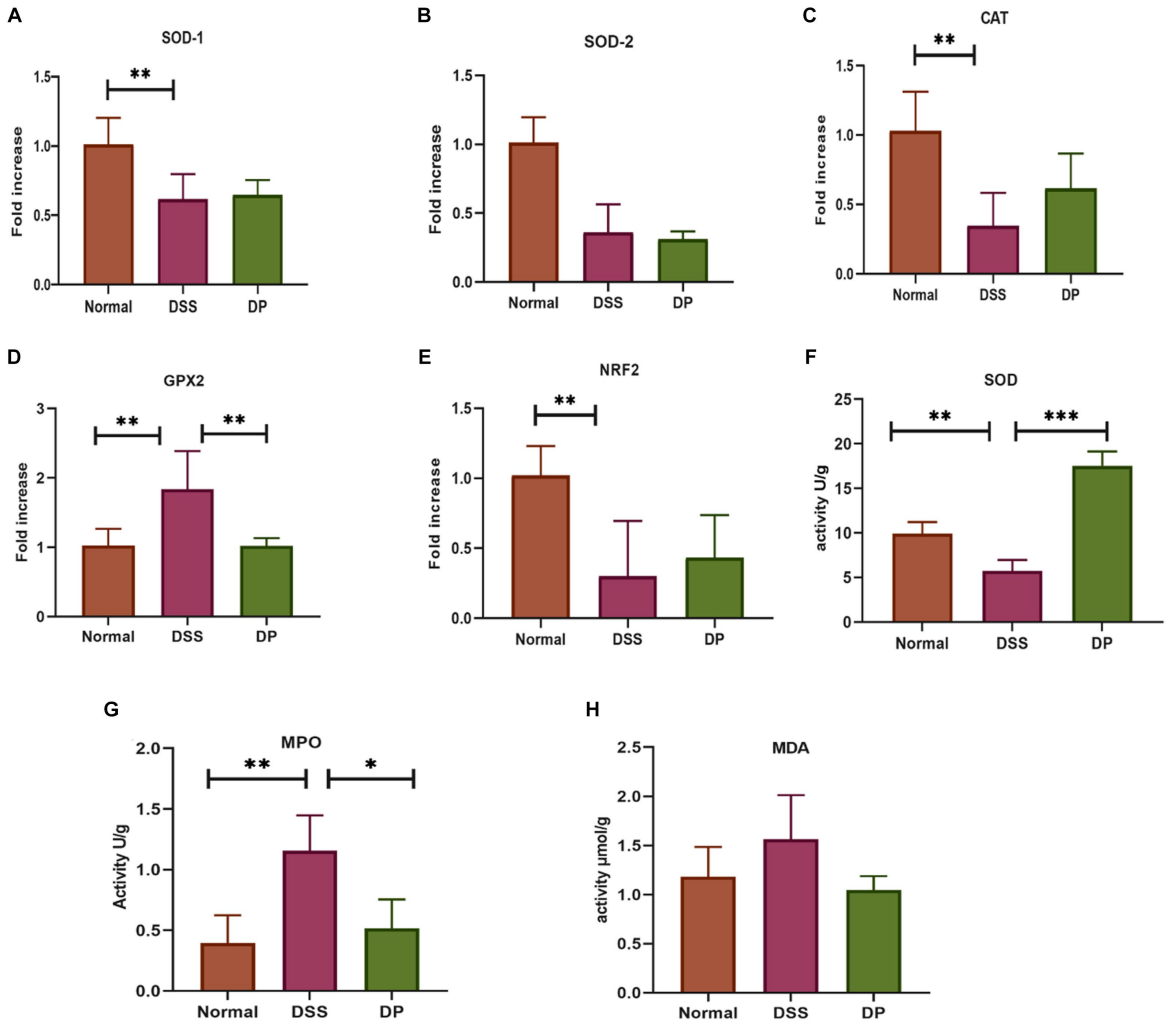
Anti-oxidant activity via RT-qPCR assessment of oxidative stress-associated genes. **(A)** Superoxide dismutase 1 (SOD-1). **(B)** Superoxide dismutase 2 (SOD-2). **(C)** Catalase. **(D)** Glutathione peroxidase 2. **(E)** Nuclear factor erythroid 2-related factor 2. **(F)** Activity of superoxide dismutase (SOD) as indicators of the antioxidant defense system. **(G)** Quantification of myeloperoxidase (MPO) activity in colonic tissue. **(H)** Levels of malondialdehyde (MDA) in colon tissue. The data were subjected to analysis of variance (ANOVA) to determine the statistical significance. The degree of significance was indicated by the notation *p = 0.05, **p = 0.001; and ***p = 0.0001, while the absence of any notation indicated lack of statistical significance (*n* = 7).

The antioxidant attributes of *L. paracasei* BNCC345679 were examined through RT-qPCR assessment of oxidative stress-associated genes including *SOD1, SOD2, CAT, GPX2,* and *Nrf2,* which unveiled its compelling antioxidant properties (Figures 2A–E). Going a step further, the SOD level was checked through ELISA, which showed *that L. paracasei* BNCC345679 intervention significantly proliferated SOD in the colon tissue as correlated with the DSS-treated group (Figure 2F). This might be the reason of the probiotic’s strong antioxidant properties, enhanced immunomodulation, or recovery of cellular homeostasis. Additionally, the impact of *L. paracasei* BNCC345679 on oxidative stress was assessed through ELISA. MPO is regarded as an important indicator of inflammatory cells, specifically neutrophil infiltration in colonic tissues. The DSS group exhibited significantly higher MPO concentrations as correlated with the normal group. Conversely, the *L. paracasei* BNCC345679-treated DP group showed significantly lower MPO concentration as correlated with the DSS group (Figure 2G). The level of MDA expression in the DSS group increased as correlated with normal whereas decreased in the DP group (Figure 2H). These results unveiled the beneficial effects of *L. paracasei* BNCC345679 in mitigating oxidative stress.

**Figure 3:**
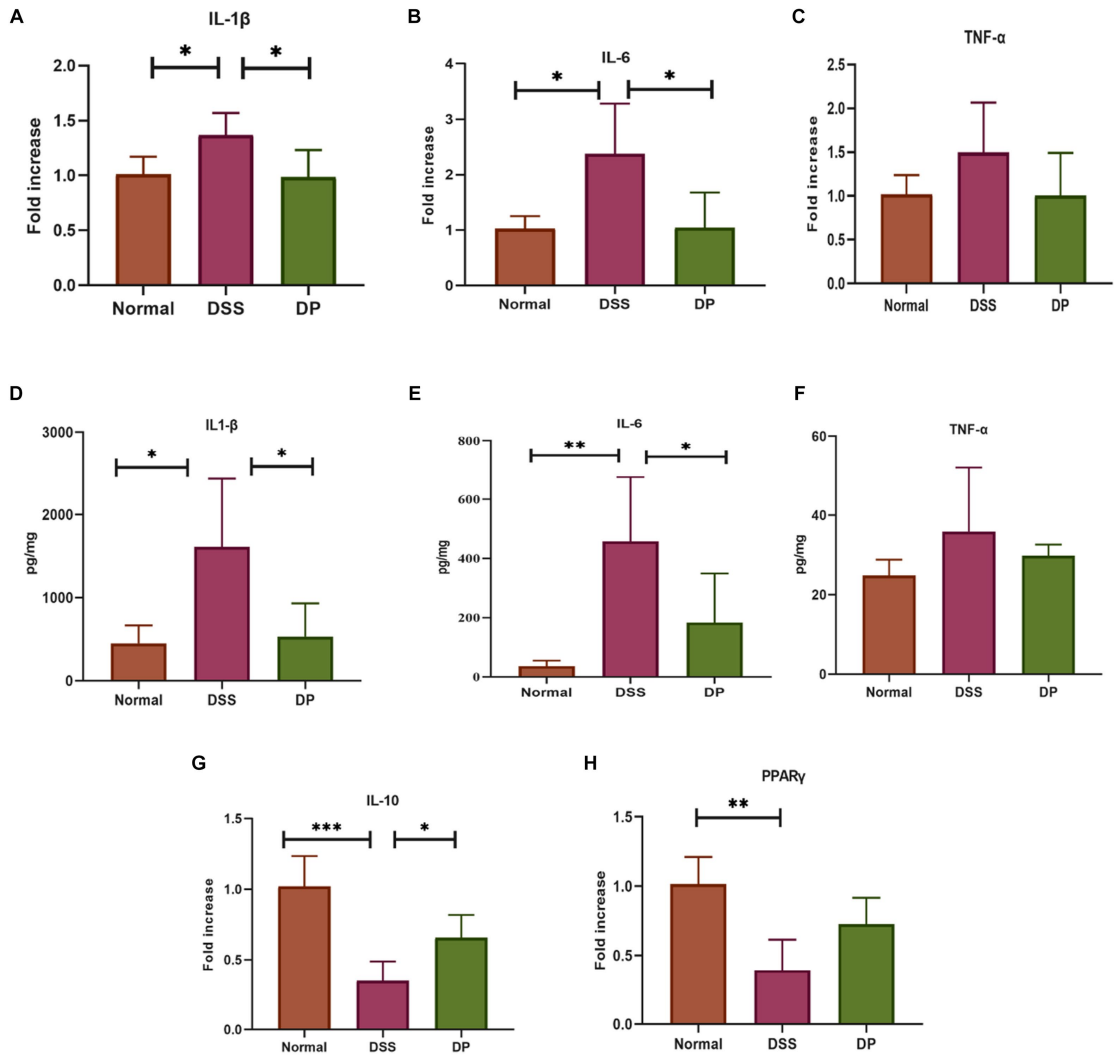
Real-time quantitative polymerase chain reaction (qPCR) analysis of pro-inflammatory cytokines. The mRNA expression of pro-inflammatory cytokines. **(A)** Interleukin-1 beta (IL-1β). **(B)** Interleukin-6 (IL-6). **(C)** Tumor necrosis factor-alpha (TNF-α). Assessment of pro-inflammatory cytokines through Enzyme-Linked Immunosorbent Assay (ELISA) to evaluate inflammatory response. **(D)** Quantification of Interleukin-1 beta (IL-1α). **(E)**. Quantification of Interleukin-6 (IL-6). **(F)** Quantification of Tumor Necrosis Factor-alpha (TNF-α). Assessment of anti-Inflammatory Cytokines through real time quantitative polymerase chain reaction (qPCR) analysis. The mRNA expression of anti-inflammatory cytokines **(G)** IL-10: Interleukin-10. **(H)** Peroxisome Proliferator-Activated Receptor Gamma (PPARα). The data was subjected to analysis of variance (ANOVA) to determine the statistical significance. The degree of significance was indicated by the notation *P=0.05, **P=0.001, ***P=0.0001, while the absence of any notation indicated lack of statistical significance. (n=7).

The transcriptional profiles of pro-inflammatory cytokine genes *TNF-α, IL-1β,* and *IL-6* were examined through RT-qPCR, which revealed that *L. paracasei* BNCC345679 intervention significantly declined the levels of cytokines. In contrast, there was a subtle or non-significant change in the level of *TNF-α* (Figures 3A–C). These molecular trends were then subsequently confirmed by ELISA examination of IL-6, TNF-α, and IL-1β in the tissues, manifesting that probiotic treatment reduced these inflammatory mediators within the DP cluster when correlate with elevated levels in the DSS cluster. However, changes in the level of TNF-α were statistically non-significant (Figures 3D–F). Furthermore, the anti-inflammatory properties of *L. paracasei* BNCC345679 were examined via RT-qPCR of anti-inflammatory genes *IL-10* and *PPARγ*. The expression of *IL-10* was significantly upregulated within the DP group, while *PPARγ* showed slight upregulation, however, which was not statistically significant (Figures 3G,H). These outcomes demonstrated that *L. paracasei* BNCC345679 also manifests anti-inflammatory properties.

### 3.3 *Lacticaseibacillus paracasei* BNCC345679 improves the expression of chosen miRNAs

**Figure 4:**
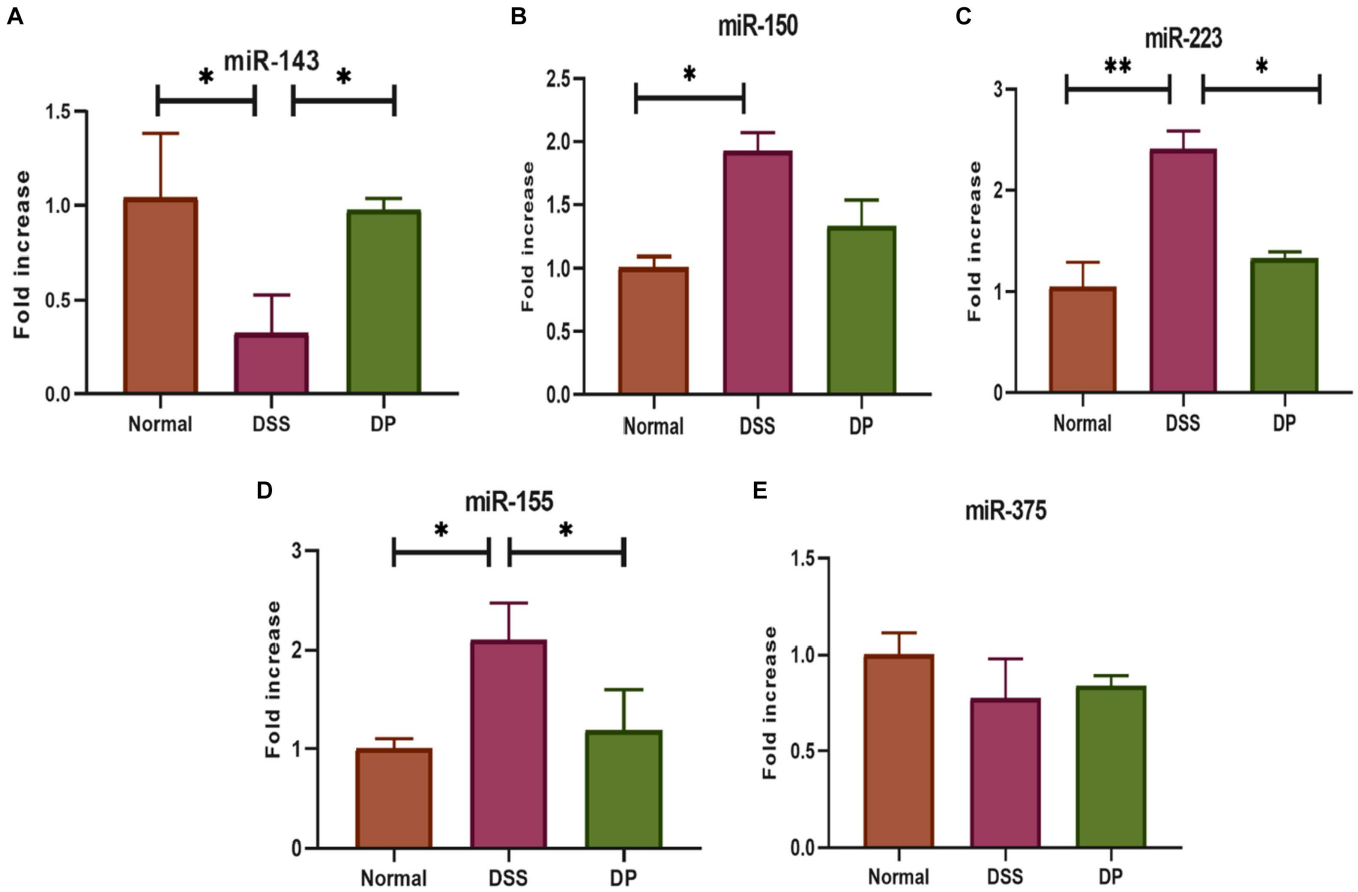
Effect of *L. paracasei* BNCC345679 on chosen miRNA gene expression using real-time quantitative polymerase chain reaction (qPCR) and was compared among different groups. **(A)** miRNA 143, **(B)** miRNA 150, **(C)** miRNA 223, **(D)** miRNA 155, and **(E)** miRNA 375. The data were subjected to analysis of variance (ANOVA) to determine the statistical significance. The degree of significance was indicated by the notation *p = 0.05, **p = 0.001, and ***p = 0.0001, while the absence of any notation indicated lack of statistical significance (*n* = 7).

Five important related miRNAs based on previous related studies were selected and evaluated by RT-qPCR in the colon. The expression levels *of miR-150, miR-155,* and *miR*-223 were significantly elevated in the DSS group than in the normal group, while *miR*-375 and *miR*-143 were downregulated. However, the expression levels of *miR*-150, *miR*-155, and *miR*-223 were significantly downregulated in the *L. paracasei* BNCC345679-treated DP group (Figures 4A–E). Moreover, the expression level of *miR*-143 was ameliorated in the DP group. In contrast, *L. paracasei* BNCC345679 treatment showed no or very little positive effect on the expression of *miR*-375 compared to the DSS-treated group.

### 3.4 *Lacticaseibacillus paracasei* BNCC345679 restores the mucus layer in DSS-induced colon

**Figure 5:**
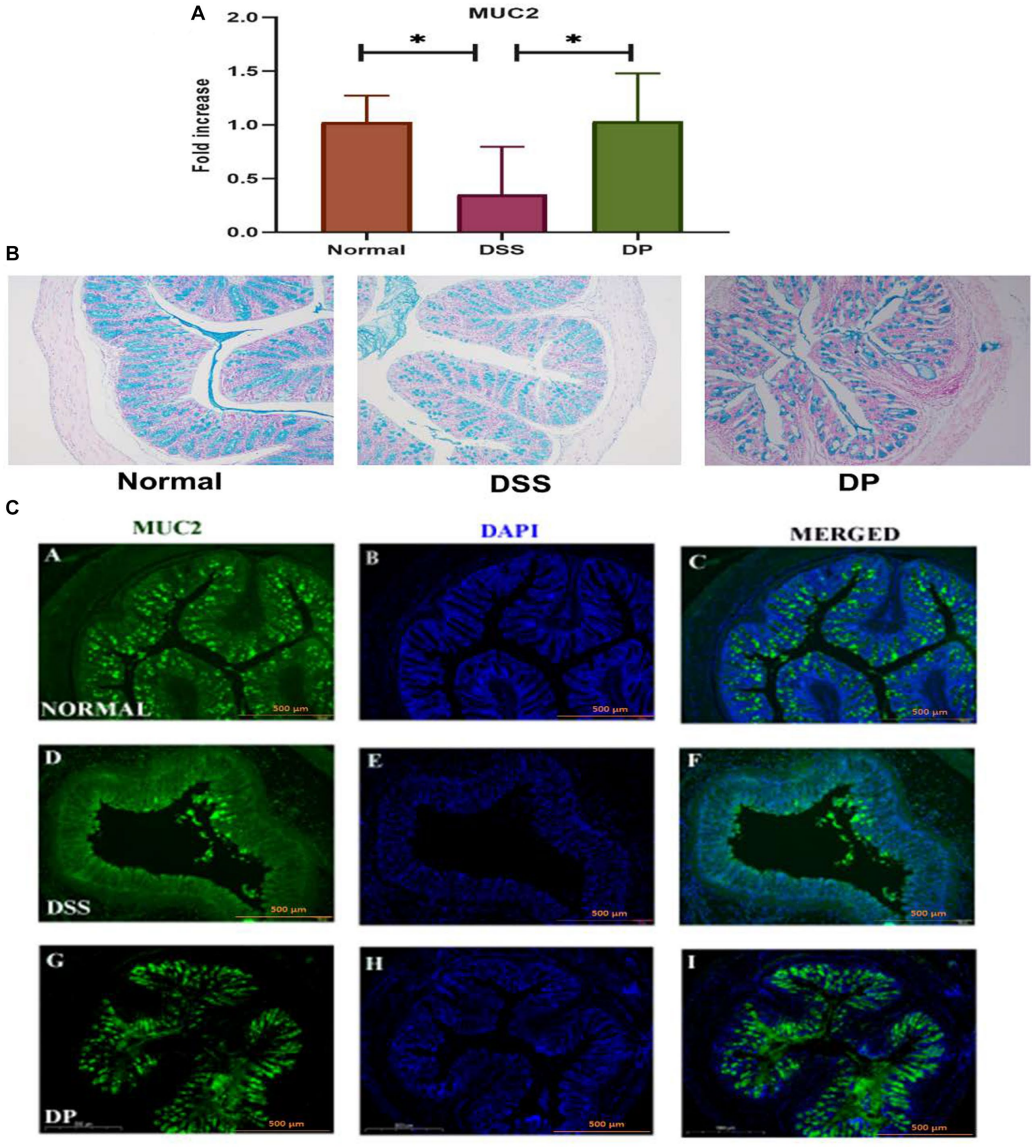
Important gene mucin 2 responsible for the formation of mucin layer was checked through RT-qPCR **(A)** MUC2 expression analysis. **(B)** Alcian blue staining of colonic tissue for mucin assessment demonstrated distinctive variations in mucin and crypt morphology among the three experimental groups. The images showed a prominent presence of mucin and intact crypt shape in the normal group, deteriorated crypt morphology and mucin depletion in the DSS group, and notable recovery of mucin content and crypt morphology in the DP group. Images presenting the prominent mucoprotective role of *L. paracasei* BNCC345679. **(C)** Representative images of immunofluorescence staining from colon sections for distribution of mucin 2. Images provided visual insight into the presence of MUC2 in each group. The normal group exhibited continues MUC2 pattern among the colonic epithelium which was deteriorated and disrupted in the DSS group. Intriguingly, the *L. paracasei* BNCC345679-treated DP group demonstrated a trend toward mucin layer restoration and MUC2 normal expression. Scale bar: 500 μm (magnification = 10x). Normal: normal control group; DSS: dextran sulfate sodium-induced colitis group; DP: *L. paracasei* BNCC345679-treated group.

The study included an aspect that how *L. paracasei* BNCC345679 administration affected the mouse’s intestinal mucus barrier. The mRNA expression of MUC2, which is a crucial element of the mucus barrier, was observed to be downregulated in mice having DSS colitis, demonstrating UC induced mucosal barrier damage and was significantly upregulated in the DP group (Figure 5A). Subsequently, Alcian blue-stained samples of the colon’s mucus layer were examined. In the DSS group, microscopic analysis indicated loss of goblet cells and damaged crypts within the epithelial lining of the colon. The mucus secretion of the colon in the DP was significantly ameliorated as correlated with DSS colitis mice (Figure 5B) with restoration of goblet cells observed. The results demonstrated a strong mucoprotective role of *L. paracasei* BNCC345679.

Eventually, immunofluorescence was employed to inspect the effects of *L. paracasei* BNCC345679 administration on the expression level of the protein MUC2. The results showed that *L. paracasei* BNCC345679 administration increased MUC2 protein expression (Figure 5C). Collectively, these results revealed that *L. paracasei* BNCC345679 administration could successfully prevent the deterioration of the mucus barrier in UC by enhancing mucin expression, especially colonic MUC2. This enhancement of the mucus barrier may potentially contribute to the anti-inflammatory attributes of *L. paracasei* BNCC345679, ultimately attenuating the symptoms and progression of ulcerative colitis.

### 3.5 *Lacticaseibacillus paracasei* BNCC345679 restores tight junction protein expression

**Figure 6:**
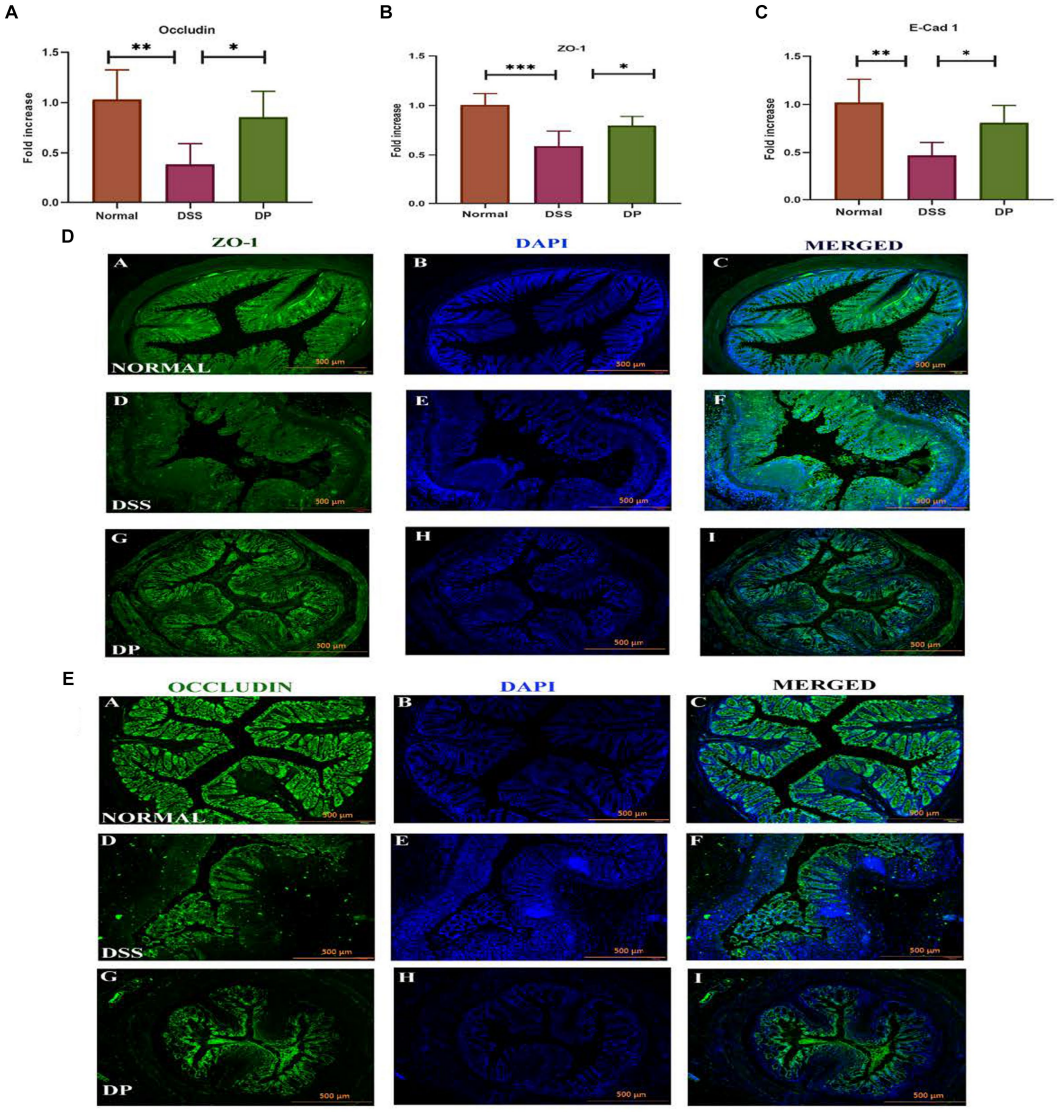
Important intestinal integrity-related genes. **(A)** Occludin, **(B)** zonula occludens-1 (ZO-1), and **(C)** E-Cadherin-1 gene expression was checked using real-time quantitative polymerase chain reaction (qPCR) and was compared among different groups. **(D)** Representative images of immunofluorescence staining from colon sections for distribution of ZO-1 involved in maintaining epithelial barrier integrity and **(E)** occludin, an important tight junction protein. Images provide insight into ZO-1 and occludin distribution and localization within the tissue of three experimental groups. The *L. paracasei* BNCC345679-treated DP group appeared to restore the concentration of both Zo-1 and occludin which is deteriorated in the DSS group showing compromised barrier function, while the normal group exhibits its undisturbed form. Scale bar: 500 μm. Normal: normal control group; DSS: dextran sulfate sodium-induced colitis group; DP: *L. paracasei* BNCC345679-treated group.

The mRNA expression of important intestinal integrity-related genes, zonula occludens-1 (ZO-1), occludin, and E-Cad-1 was carried out which revealed that the DSS model group was severely affected as ZO-1, occludin, and E-Cad-1 were significantly downregulated compared to the normal group while found to be increased and recovered in the DP-treated group (Figures 6A–C). In due course, immunofluorescence investigation for ZO-1 and occludin protein expression revealed a compact colonic structure in normal mice, supporting the role of ZO-1 and occludin in preserving the integrity of the epithelial barrier. However, a deteriorated colon was marked in the DSS-treated group, indicating that the protein normal function had been interfered, whereas the compactness of colon structure has been significantly recovered in the DP-treated group (Figures 6D,E). Overall, the data supported that DSS-treated mice had lower ZO-1 and occludin expression, which was recovered within the DP group.

### 3.6 *Lacticaseibacillus paracasei* BNCC345679 modulates VCAM-1 expression

**Figure 7:**
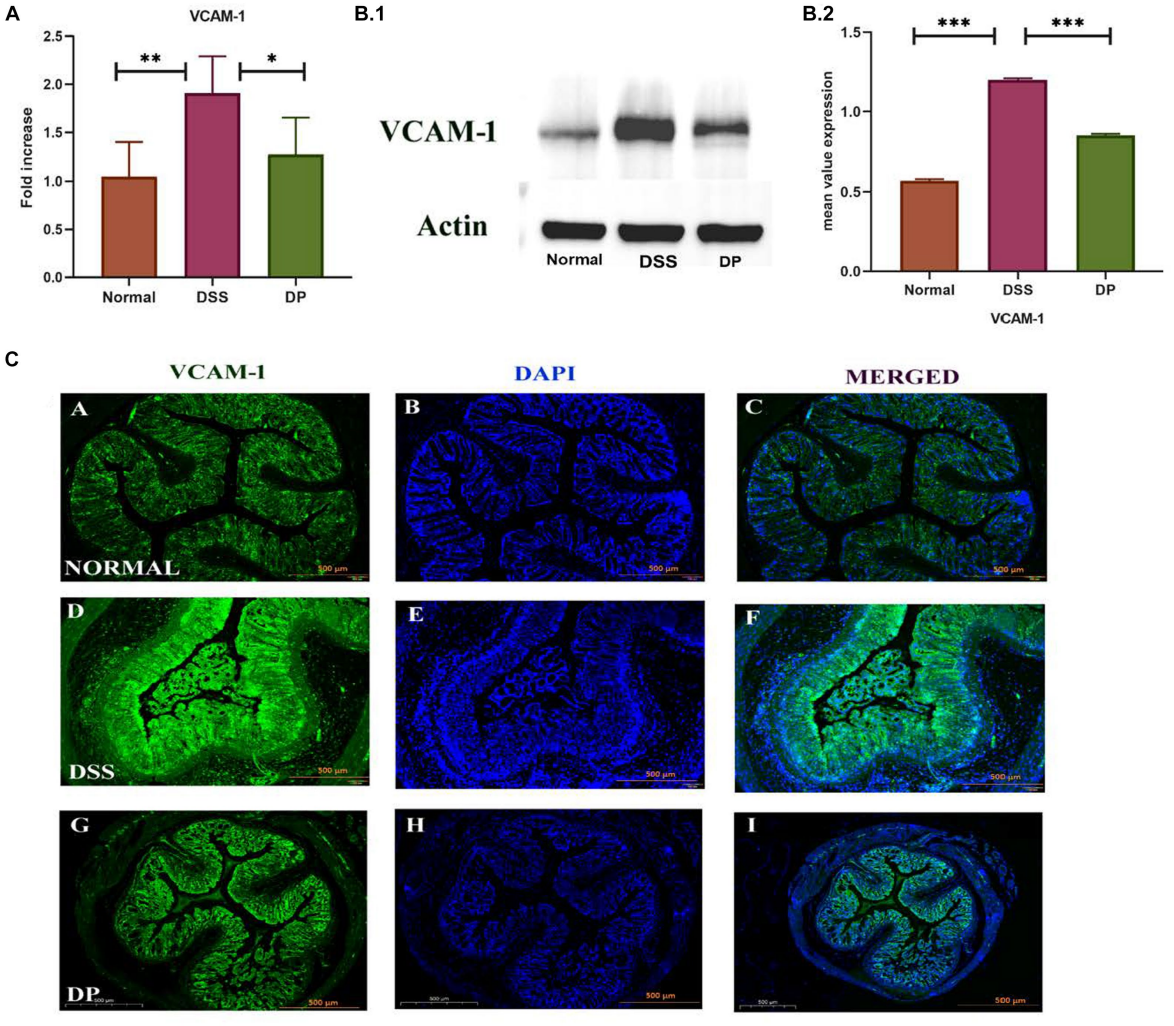
**(A)** VCAM-1, also known as vascular cell adhesion molecule-1. The examination of the expression levels was conducted using the real-time quantitative polymerase chain reaction (qPCR) technique. **(B,C)** Western blot analysis was employed to examine the protein expression levels of VCAM-1 in colonic tissues among the three experimental groups. The normal group demonstrated lower level of VCAM-1 expression which is elevated in the DSS group indicating inflammation. In contrast, the DP group exhibited ameliorated VCAM-1 expression suggesting a potential anti-inflammatory effect of *L. paracasei* BNCC345679. **(C)** Representative images of immunofluorescence staining from colon sections to evaluate VCAM-1 expression. The normal group demonstrated minimal VCAM-1 staining, while the DSS group displayed enhanced VCAM-1 expression speculating inflammation. In contrast, the DP group exhibit reduced VCAM-1 fluorescence indicating a potential amelioration of inflammation due to *L. paracasei* BNCC345679 intervention. Scale bar: 500 μm. Normal: normal control group; DSS: dextran sulfate sodium-induced colitis group; DP: *L. paracasei* BNCC345679-treated group.

To study the implication of *L. paracasei* BNCC345679 on adhesion molecules, i.e., vascular cell adhesion molecule-1 (VCAM-1), RT-qPCR, Western blot, and immunofluorescence were carried out on colonic tissue samples from all three groups. It was observed that in the normal group, the expression of VCAM-1 was lower compared to both other groups. The DSS model group showed a significant upregulation of VCAM-1 expression compared to the normal group. However, in the DP group, VCAM-1 was downregulated significantly as correlated with the DSS model group (*p* < 0.001; Figures 7A–C). These findings suggest that *L. paracasei* BNCC345679 administration may serve as a potential strategy for alleviating inflammation and mitigating the symptoms of DSS-induced colitis.

### 3.7 *Lacticaseibacillus paracasei* BNCC345679 administration significantly alters microbial community structure

**Figure 8:**
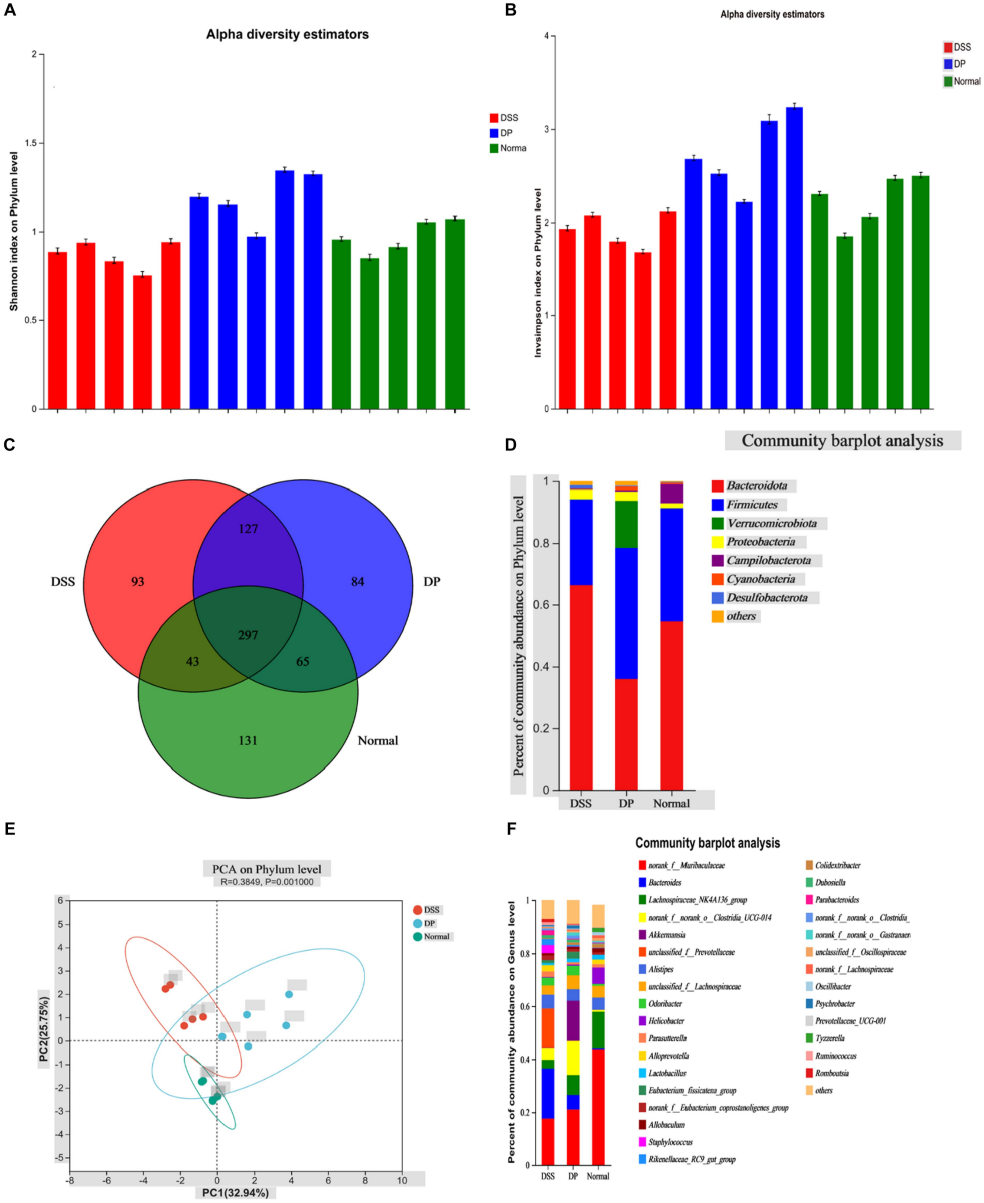
Effect of *L. paracasei* BNCC345679 on gut microbiome diversity in DSS-induced colitis. **(A)** Alpha diversity metrics, Shannon index on the phylum level demonstrated that the DSS group is least divers among the three experimental groups, while remarkably DP is highly diversifies group. **(B)** Alpha diversity InvSimpson index on the phylum level confirmed the previous result trend. **(C)** All three groups having shared and unique OTUs. **(D)** Microbial taxa structure is significantly varied among the three experimental groups as per community bar plot analysis on the phylum level. **(E)** Principal component analysis plot of fecal microbiota structure based on Bray–Curtis distances. **(F)** Community bar plot analysis on the genus level showed relative bacterial abundance on the genus level (*n* = 5).

The effect of *L. paracasei* BNCC345679 on gut microbiome was investigated using high-throughput sequencing. Microorganism variety was significantly lowered in the DSS group when correlated with the normal group. Furthermore, this gut microbiome diversity was enhanced in the *L. paracasei* BNCC345679-treated DP group (Figures 8A,B). As can be observed, there were variations in the patterns of gut microbiota among the three groups. All three groups were having shared as well as unique OTUs (Figure 8C). Community bar plot analysis demonstrated discernible variations in gut microbiota structure among the three groups. A disruption in the gut microbiota was induced by DSS therapy; however, it was lowered when the mice were treated with *L. paracasei* BNCC345679. The most plentiful phyla in the analyzed groups were *bacteroidota* followed by *Firmicutes.* However, at the phylum level, considerable variations in microbial composition were found among the three groups (Figure 8D). When compared to control mice, the DSS group had much more members of the phylum *Bacteroidota;* however, this rise was able to be attenuated by *L. paracasei* BNCC345679 treatment. Phylum *verrucomicrobiota* was also significantly higher in the DP group when correlated with both the normal and DSS colitis mice groups. The normal group had the highest amount of *Campylobacterota* among all three study groups.

Utilizing the Bray–Curtis complementary algorithm to make an ordination plot of the distance matrix, a clear separation between bacterial communities originating from the normal and DSS groups was visible. This separation was markedly within the principal coordinate analysis (PCA) plot (Figure 8E). At the genus level, the prominent microbiota in the normal group were *Muribaculaceae,* followed by *Lachnospiraceae* and *Alistipes*. On the contrary, *Muribaculaceae* and *Lachnospiraceae* were significantly lowered in the DSS-treated group while *Bacteroides* was relatively the most abundant genus here. Unclassified *Prevotellaceae* and *Bacteroides* were notably lowered in the DP group correlated with the DSS mice group. Notably, the amount of *Akkermansia* was significantly enhanced in the probiotic group (DP) compared to both other groups. Moreover, member of the genus *Clostridia* was increased as a result of *L. paracasei* BNCC345679 treatment. The normal group revealed the highest amounts of genus *lactobacillus* that were notably decreased by DSS treatment in the DSS-treated group and significantly recovered by *L. paracasei* BNCC345679 treatment in the DP group (Figure 8F).

## 4 Discussion

Dysbiosis of the intestinal microbiome, differential expression of miRNA, and alterations in tight junction proteins potentially contribute to IBD induction. Moreover, oxidative stress genes play a remarkable role in colitis (Giustarini et al., 2009). Unfortunately, traditional pharmacological therapies are not potentially used for treating IBD amid susceptibility and severe side effects (Kotlyar et al., 2015). Therefore, biomedicine-based pharmacotherapies such as probiotics have been used as an alternative for treating IBD (Veerappan et al., 2012). Probiotics hold an essential part in the modulation of the immune system, homeostasis of the microbial host, and the integrity and functionality of the epithelial barrier. *Lactobacillus* has been investigated worldwide due to its valuable effects in the mitigation of intestinal inflammation in individuals with IBD and animal models (Jang et al., 2019). In this investigation, with the help of *in vivo* DSS model, we showed that a *Lacticaseibacillus paracasei* strain BNCC345679 altered the immune reaction and reduced the manifestation of IBD and can modulate the combination of various cytokines. The probiotic stimulation raises *IL-10* and *PPARγ* extravasation, which is essential for anti-inflammatory reaction. Moreover, it downregulated the expression of pro-inflammatory genes, namely, *TNF-α, IL-1β*, and *IL-6,* which coincide with previous related studies (Dong et al., 2022).

It is a valuable model to study the clinical efficacy and potential underlying operative mechanisms of how probiotics affect UC. Mice treated with 3% DSS for 1 week showed all UC-related clinical manifestations (Chassaing et al., 2014). In the context of our research, oral gavage of *L. paracasei* BNCC345679 significantly inhibited weight loss and improved DAI score. Furthermore, colon structure and length, along with mouse weight, were notably improved in the DP group compared to the DSS group. In addition, *L. paracasei* BNCC345679 improved mucosal crypt structure, goblet cells, and colon compactness as shown in the histological analysis by improving the expression of MUC2 and tight junction protein, i.e., ZO-1 and occludin. Alterations in tight junction protein expression and distribution are key to UC development and progression (Al-Sadi et al., 2014). In addition, the administration of *L. paracasei* BNCC345679 lowered the innate immune reaction as consents to previous findings (Hall et al., 2011). Reductions in MPO levels indicated amelioration of oxidative stress, changes in neutrophil infiltration, and colonic tissue damage (Eiserich et al., 1998). Reduction in the level of MPO in the DP group might have contributed to the anti-inflammatory properties. In addition, inflammatory cytokines, notably TNF-α, accompanied by T-cells, are synthesized by macrophages that take part in the pathogenesis of IBD. Moreover, TNF-α activates the NF-κB regularity pathway that collectively stimulates cytokines. On the contrary, the *PPARγ* downregulates the activation of NF-κB (Fischer et al., 2013). In this study, *L. paracasei* BNCC345679 upregulated *PPARγ* and *IL-10.* Additionally, it downregulated the expression of *IL-1β*, *IL-6*, and *TNFα* in the *L. paracasei* BNCC345679*-*treated group.

VCAM-1 is a cell adhesion molecule (CAM) that plays a pivotal role in leukocyte attraction, recruitment, and adhesion during inflammation. Because of inflammatory stimuli, its expression is upregulated, leading to the facilitation of leukocyte adhesion to endothelial cells in the colonic venules of DSS colitis mice (Soriano et al., 2000). Another study suggests that VCAM-1 serves as an essential intermediary that sustains and enhances the inflammatory procedure by facilitating the recruitment of leukocytes to the site of inflammatory activity in colitis (Sans et al., 1999). Our findings are parallel to previous studies as VCAM-1 was upregulated in the DSS group as compared to the normal group but was significantly downregulated by probiotic administration. These results suggest that *L. paracasei* BNCC345679 actively modulates the host immune response to counteract inflammation in colitis. Moreover, *L. paracasei* BNCC345679 intervention compellingly restored mucin and goblet cell production that was deteriorated by DSS administration. MUC2 and ZO-1 play a pivotal role in protecting intestinal epithelium, ensuring barrier integrity and the substantial decrease in MUC2, ZO-1, and goblet cell levels in the DSS-treated group were a clear indication of compromised mucosal defense. In blunt contrast, *L. paracasei* BNCC345679 intervention significantly upregulated MUC2 and ZO-1 levels and restored goblet cell production to normalcy. These results strengthen the notion that *L. paracasei* BNCC345679 can augment the protective mucin layer. Our results also validated some previous related studies (Johansson et al., 2011; Ren et al., 2018).

The modulation and expression of tight junction proteins’ microRNAs and the consequent variation in epithelial barrier function and inflammation in IBD are well recognized. Correspondingly, variation in the level of microRNAs of healthy and IBD patients revealed their role in disease onset (Xiao and Rajewsky, 2009). Pro- and anti-inflammatory signals, including cytokines, influence the expression of these microRNAs. It has been reported that *miR*-155 upregulates in macrophages as well as in B and T lymphocytes (O’Connell et al., 2010, 2012). Another study has found that *miR*-155^−/−^ murine model has natural protection from DSS colitis by upregulation of Th1/Th17 (Singh et al., 2014). More studies have confirmed that *miR*-155 and *miR*-150 modulate the immune response by governing the differentiation and development of innate immune cells and are upregulated in UC mice (Bian et al., 2011). The present study has also verified this upregulation, and *L. paracasei* BNCC345679 has successfully downregulated them and yielded positive immunomodulatory effects in the DP group. Furthermore, *miR*-143 plays a role in preserving normal colonic biology. It has been observed to be downregulated in DSS colitis, which results in the appearance of pro-inflammatory cytokines (Starczynowski et al., 2010). *L. paracasei* BNCC345679 was able to significantly restore it in our study, which suggests the restoration of colonic function. *miR*-223 has been documented to exhibit significant upregulation in UC mice and is implicated in the production of IL-1β and inflammasome complex (Haneklaus et al., 2012). Our probiotic intervention demonstrated a notable reduction in *miR*-223 expression, speculating that it may be potentially involved in downregulating the expression of IL-1β levels within the DP group. Moreover, *miR*-375 is thought to be a multitasking and versatile miRNA, involved in different biological processes, i.e., regulation of glucose, development of pancreatic islet carcinogenesis, and cell differentiation (Xu et al., 2011). In our investigation, *miR*-375 was downregulated significantly in DSS-treated mice as correlated with the normal group; however, *L. paracasei* BNCC345679 did not prove to be effective in the upregulation of *miR*-375. It is worth highlighting that investigating the effects of probiotics on miRNA is in the initial phase, and a limited number of investigations have been conducted in this regard. Although our study provided valuable insight into the effect of probiotics on colitis-related miRNA modulation, there was a limited scope of miRNA analysis; a broader miRNA profiling approach such as microarray or next-generation sequencing can potentially reveal a more comprehensive understanding of the regulatory networks behind it. Additionally, there was a lack of miRNA functional validation role, miRNA–mRNA interaction, and a mechanistic links between miRNA and other observed effects. Furthermore, studies aided with omics of miRNA and mRNA investigation in human cell culture models will further increase the understanding of its mechanistic role in clinical relevance. However, our results suggest that *L. paracasei* BNCC345679 intervention in DSS colitis murine model might ameliorate the dysregulated immune response possibly by modulating the expression of miRNA.

The influence of the gut microbiota on the immune system has recently attracted considerable scholarly interest. The growing research shows that the gut microbiota has an important part in mitigating ulcerative colitis and can direct the reduction in microbial diversity resulting in significant alteration of microbiome composition. Our findings revealed that DSS-induced colitis has altered microbial composition and curtailed the variability in gut microbiota. Nevertheless, in line with past studies, *L. paracasei* BNCC345679 administration restored gut microbiota diversity and composition. Gut microbiome composition was evaluated at both the phylum level and the genus level. *Bacteroides* were observed to be increased in the DSS-treated group while not in abundance in the normal group. However, their abundance was lowered by *L. paracasei* BNCC345679, suggesting their role in ameliorating DSS colitis, boosting recovery, and reshaping the gut microbiome. In line with our findings, previous investigation has also demonstrated the increased abundance of *Bacteroides* in the DSS-treated group (Almagro-Moreno and Boyd, 2009; Schwab et al., 2014; Jang et al., 2019). There may be possible signals of UC onset in the murine DSS model, while their decrease may suggest the recovery phase. Consistent with previous studies (Liu et al., 2021; Wu et al., 2023), beneficial *norak_f_Muribaculaceae* were found to be highest in abundance in the BPS-treated normal group while they were significantly decreased in the DSS-treated group. However, *L. paracasei* BNCC345679 intervention noticeably recovered them. Increased abundance of *Lactobacillus* was observed in the DP group treated with DSS and *L. paracasei* BNCC345679 compared to the group only treated with DSS. In a similar manner, the DP group also exhibited increased abundance of *Akkermansia* correlated with the DSS group. *Lactobacillus* promotes the assembly of *IL-10* by stimulating DCs (Mohamadzadeh et al., 2011). *Akkermansia* drives anti-inflammatory effects, which reduce inflammation through different activities (Shin et al., 2014). Furthermore, *Akkermansia* prompts the assembly of interleukin-10 and aids in preserving gut homeostasis and enhancing barrier function (Ottman et al., 2017). The oral intervention of *L. paracasei* BNCC345679 may potentially help restore *Akkermansia* and *Lactobacillus* population, which may drive an increase in *IL-10* levels, positively affect the cohesion of the mucus layer, and maintain gut homeostasis, which may relieve DSS-induced colitis. Studies have revealed that probiotic intervention can affect the diversity of specific gut microbiota in a short time of up to 24 h via dietary changes (Wu et al., 2011; Sun et al., 2018; Aziz et al., 2023). The effects of probiotics have been widely investigated and have yielded some promising results. However, it is important to remember that these probiotics’ effects vary mostly depending on the strain (de Roock et al., 2010; Hua et al., 2010).

These bacteria in the gut are metabolically very much interdependently networked through their metabolic pathways and products. Thus, further evaluation is needed to study the particular function of various microbial taxa and the production of its specific metabolites within the gut.

## 5 Conclusion

This study identified and investigated the therapeutic potential of *L. paracasei* BNCC345679 to ameliorate DSS-induced colitis by affecting immune cytokine expression, tight junction protein, and gut microbiome. Given its promising properties, we suggest that *L. paracasei* BNCC345679 exhibits therapeutic potential and may guide us toward a novel approach in manipulating the gut microbiota to control UC.

## Data availability statement

The original contributions presented in the study are included in the article/Supplementary material, further inquiries can be directed to the corresponding author.

## Ethics statement

The animal study was reviewed and approved by the Bioethical Committee of Southwest Medical University, Luzhou, under the approval number: SWMU 202/0049. The studies were conducted in accordance with the local legislation and institutional requirements. Written informed consent was obtained from the owners for the participation of their animals in this study.

## Author contributions

WA: Data curation, Formal analysis, Writing – original draft. AD: Conceptualization, Writing – review & editing. TK: Validation, Writing – original draft. MR: Data curation, Investigation, Writing – original draft. AH: Investigation, Software, Writing – review & editing. TA: Formal analysis, Visualization, Writing – review & editing. MA: Data curation, Software, Writing – review & editing. JW: Funding acquisition, Resources, Supervision, Visualization, Writing – review & editing.

## Footnotes

^1^ https://cloud.majorbio.com
