# Supplementary material for: Lacticaseibacillusparacasei BNCC345679 revolutionizes DSS-induced colitis and modulates gut microbiota
Source: Front Microbiol. 2024 Mar 27;15:1343891. doi: 10.3389/fmicb.2024.1343891 (PMC11004379; doi:10.3389/fmicb.2024.1343891)
Supplement: Supplementary file 1 [file Table_1.DOCX]

| Assigned Scores | Rectal Bleeding | Rectal Prolapse | Diarrhoea | Colonic bleeding |
| --- | --- | --- | --- | --- |
| 0 | None | None | No signs | No signs |
| 1 | Red | Mild | Soft stools | Red |
| 2 | Dark red | Higher | Very soft | Dark red |
| 3 | Gross bleeding | Extensive | Watery stools | black |

**Table S1: Standard Score Scale for Disease activity index DAI**

**Table S2: Histological Standard Score for H&E Stain Images Analysis**

*Scores shown is an average for each animal

| Assigned Score | Inflammation | Mucosal injury | Crypt damage | Lesion area (%) |
| --- | --- | --- | --- | --- |
| 0 | Absent | Absent | Absent | Absent |
| 1 | Signs | Mucous layer | 1/4 | 1-20 |
| 2 | Mild | Submucosa | 1/3 | 21-40 |
| 3 | Moderate | Muscularis | 2/3 | 41-70 |
| 4 | Severe | Muscularis and serosa | 80% | 80 |

**Table S3. Primers Used in This Study**

| **Gene** | **Sequence (5’-3’)** | **Annealing temperature (ºC)** |
| --- | --- | --- |
| *IL-1β* | FW: CTGAACTCAACTGTGAAATGC  RV: TGATGTGCTGCTGCGAGA | 55 |
| *TNF-α* | FW: AGGGTCTGGGCCATAGAACT  RV: CCACCACGCTCTTCTGTCTAC | 60 |
| *IL-6* | FW: CCCTTTGCTATGGTGTCCTT  RV: TGGTTTCTCTTCCCAAGACC | 60 |
| *IL-10* | FW: CCCTTTGCTATGGTGTCCTT  RV: TGGTTTCTCTTCCCAAGACC | 60 |
| *PPARγ* | FW: CAGGCTTGCTGAACGTGAAG  RV: GGAGCACCTTGGCGAACA | 62 |
| *MUC-2* | FW: ATGCCCACCTCCTCAAAGAC  RV: GTAGTTTCCGTTGGAACAGTGAA | 56 |
| *E-Cad 1* | FW: CAGGTCTCCTCATGGCTTTGC  RV: CTTCCGAAAAGAAGGCTGTCC | 60 |
| *Claudin 3* | FW: AACTGCGTACAAGACGAGACG  RW: ATCCCTGATGATGGTGTTGG | 61 |
| *ZO-1* | FW: CTTCTCTTGCTGGCCCTAAAC  RV: TGGCTTCACTTGAGGTTTCTG | 56 |
| *SOD1* | FW: TAACTGAAGGCCAGCATGGGT  RV: GGTCTCCAACATGCCTCTCTTC | 55 |
| *SOD2* | FW: CAGACCTGCCTTACGACTATGG  RV: GCTGAAGAGCGACCTGAGTTGT | 55 |
| *VCAM-1* | FW: TGGTGAAATGGAATCTGAACC  RV: CCCAGATGGTGGTTTCCTT | 55 |
| *CAT* | FW: TTGTTCAGTGACCGAGGGATT  RV: TTCCTGAGCAAGCCTTCCTG | 55 |
| *Nrf2* | FW: ACTTCGGTGATTCTGTTAG  RW: CTCGTGCTCGCTTACT | 55 |
| *Occludin* | FW:ATTCCATCAGTTTCCTATCT  RV:ACCAGGACCTTTCTTGAC | 49 |
| *GPX2* | FW: GAACGAGGAGATCCTGAACAGC  RV: GGTAGGGCAGCTTGTCTTTCAG | 55 |
| *GAPDH* | FW: GTATGACTCCACTCACGGCA  RV: GATGTTAGTGGGGTCTCGCT | 60 |
